# Application of a robotic THz imaging system for sub-surface analysis of ancient human remains

**DOI:** 10.1038/s41598-019-40211-7

**Published:** 2019-03-04

**Authors:** Eva-Maria Stübling, Arno Rehn, Tabea Siebrecht, Yannick Bauckhage, Lena Öhrström, Patrick Eppenberger, Jan C. Balzer, Frank Rühli, Martin Koch

**Affiliations:** 1 Department of Physics and Material Science Center, Philipps-Universität Marburg, Renthof 5, 35032 Marburg, Germany; 2 Center for Optical Technologies, Aalen University, Center for Optical Technologies, Anton Huber Strasse 21, 73430 Aalen, Germany; 3 Institute of Evolutionary Medicine, Faculty of Medicine, University of Zurich, Zurich, Switzerland; 4 Universität Duisburg-Essen, Bismarckstraße 81, 47057 Duisburg, Germany

## Abstract

We used a robotic-based THz imaging system to investigate the sub-surface structure of an artificially mummified ancient Egyptian human left hand. The results obtained are compared to the results of a conventional CT and a micro-CT scan. Using such a robotic THz system promises new insights into the sub-surface structure of human remains. The depth resolution of the THz images exceeds the resolution of a conventional CT scan and is comparable with a micro-CT scan. The advantage of THz measurements over micro-CT scans is the fact that even comparatively large samples, like complete bodies, can be scanned. These would not fit into a conventional micro-CT scanner.

## Introduction

Ancient human remains are very valuable for the study of the evolution of mankind and disease^1–4^. Hereby, mummified remains are of special interest, since the preserved soft tissue allows for the investigation of a wide spectrum of disease^5–8^. For the investigation of such precious remains non-invasive methods are desirable. Currently the gold-standard are radiological investigations by CT and X-ray.

Terahertz (THz) time-domain imaging (TDI) and spectroscopy (TDS) are relatively new techniques in the research field of paleo-pathology. In comparison to well-established techniques such as conventional X-ray and X-ray computed tomography (CT)^9,10^, THz time-domain imaging was first demonstrated in 1995^11^. Due to its potential in non-invasive material testing^12–15^ with a depth resolution in the submillimeter range^16–19^, THz TDI has aroused much interest in recent years. The interaction mechanisms of matter with X-ray radiation are fundamentally different from those with THz waves. While X-rays have photon energies in the keV range THz waves have photon energies of just a few meV. X-ray photons are absorbed by kicking electrons from the electron shell of the atoms constituting the sample. The magnitude of this effect scales with the atomic number of the elements. Besides X-ray photons can lose energy by the so called Compton effect, when part of the energy of the photon is transferred to a recoiling electron. In addition, elastic scattering (Thomson scattering, Rayleigh scattering) is possible, which also attenuates the intensity of an X-ray beam traveling through a medium. In contrast, THz waves possess very little photon energy. They are scattered elastically as well but they do not contain enough energy to ionize an atom^20^, which simplifies its application in field studies since no special radio-protection is necessary. Instead they excite intramolecular of intermolecular vibrations in dry biological samples. In the THz frequency range many non-polar materials are highly transparent and several organic molecules show characteristic spectroscopic features^21^. Thus, THz imaging represents different material properties than radiographic imaging modalities such as conventional X-ray or CT.

In recent years different attempts were made to apply THz TDI in heritage science^22–24^. Simultaneously THz TDS systems, which allow a fast and accurate imaging process with a high signal to noise ratio, became commercially available^25,26^. Often THz TDI is realized using a raster scan scheme. Therefore typically a 2D translation unit is used, moving either the THz sensor unit or the sample in the focal plane. Based on the obtained data, a tomographic reconstruction of the imaged sample can then be performed visualizing its internal structure^27–29^. For thick or highly absorbing samples, a reflection configuration must be used. Until recently measurements in reflection configuration were limited to planar samples, since radiation should be incident from a defined distance and at a perpendicular angle to the specimen’s surface. By combining a robotic arm with a THz TDS system, it recently became possible to overcome this limitation and to also investigate samples of complex three-dimensional shape^30^. First THz TDI measurements have shown the potential of THz radiation in the field of mummy research^31–34^. Using a robotic THz TDI system for the investigation of ancient human remains promises new insights into their sub-surface structure, which is presented in this article. Furthermore, THz TDI imaging data were compared to CT and Micro-CT imaging data for morphological correlation.

## Results

### Investigated sample

**Figure 1:**
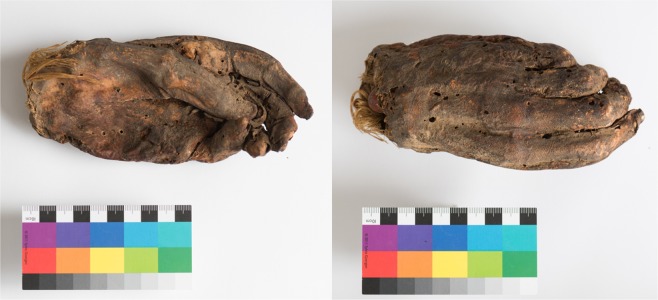
Investigated ancient Egyptian artificially mummified hand (Photographs with kind permission of the Institute of Evolutionary Medicine, University of Zurich, photographer Corina Steiner (CC BY open access license)).

The sample investigated is an artificially mummified ancient Egyptian human left hand from the mummified human tissue collection of the Institute of Evolutionary Medicine (ex-collection of the Musee d’Orbe, Switzerland, radiocarbon dated to approx. 1500–1100 BCE^31^), shown in Fig. 1.

This study was conducted with institutional review board approval (BASEC N° 2017-00260, “Correlative radiological, histological, bacteriological and molecular assessment of modern and ancient embalming methods for human tissue.”) and in accordance with the code of ethics of the Institute of Evolutionary Medicine of the University of Zurich^35^, which demands a careful judgment of the appropriateness of any research involving ancient human remains against the applied degree of invasiveness. There was no financial support from the industry for this study.

### Robotic THz system and data processing

The sample is investigated by using a robotic-based THz system^30^. With this scheme the THz emitter and receiver can be positioned perpendicular and at defined distance to the sample surface enabling the measurement of samples with a complex surface. The system achieves information about the sample by measuring a three-dimensional mesh of points based on the surface of the sample. Therefore, first the shape of the surface has to be acquired.

**Figure 2:**
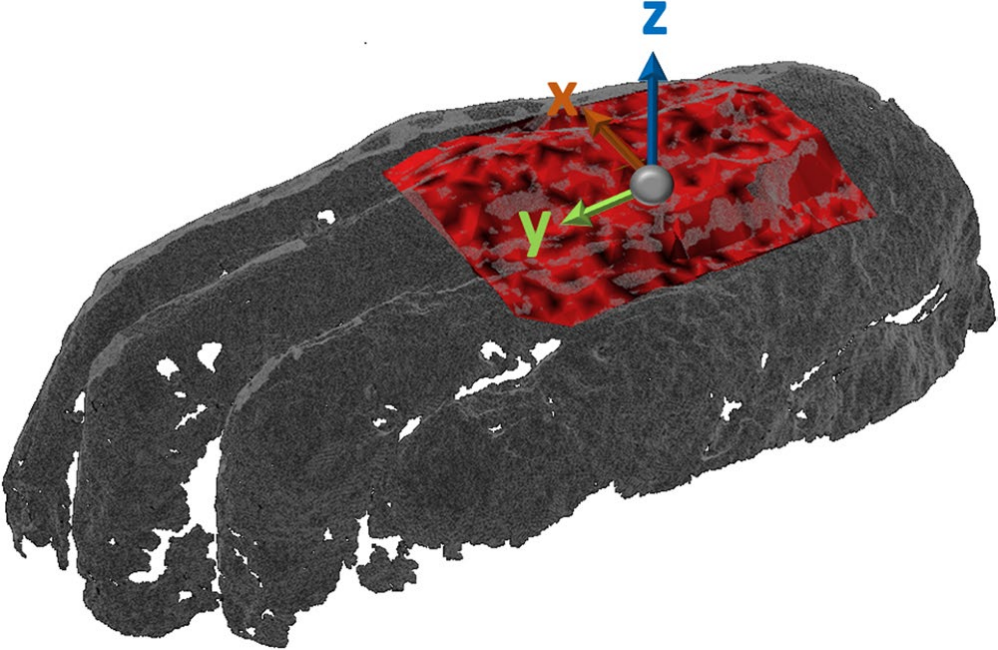
3D-Scan of the investigated mummy hand. The measurement area is highlighted in red.

Figure 2 shows the three-dimensional surface of the investigated mummy hand obtained with a structured light scanner system. The THz measurement area is highlighted in red. The color intensity of the red region encodes the peak to peak amplitude of the deconvolved signal of the first reflected THz pulse.

**Figure 3:**
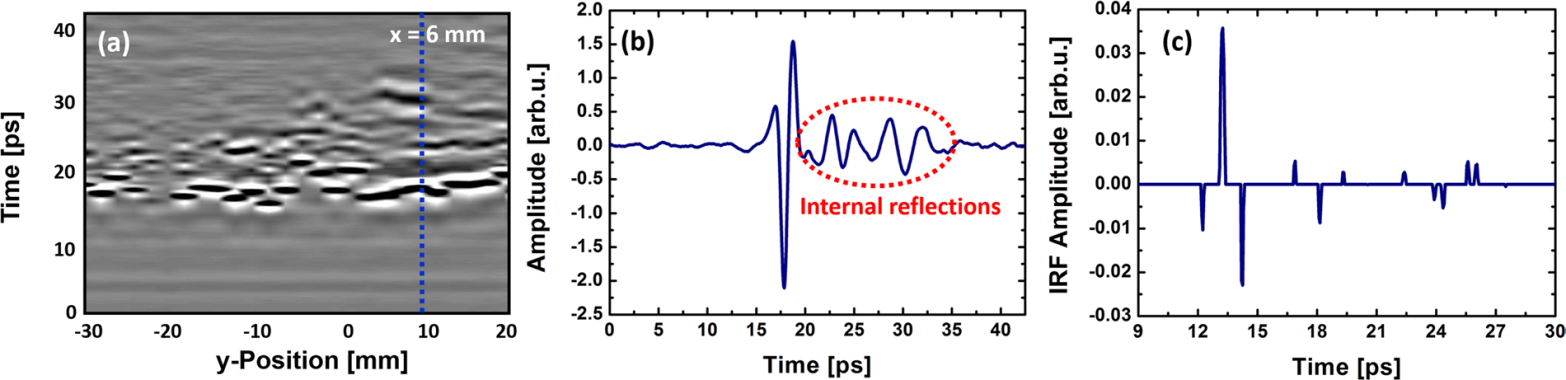
(**a**) Line scan at x = 6 mm. (**b**) Exemplary THz time trace at x = 6 mm and y = 10 mm, indicated by the blue line in (**a**). (**c**) Impulse response function of the deconvolved THz pulse shown in (**b**).

To illustrate the principle of the THz tomography measurements a line scan for a constant x-position and parallel to the y-axis of the obtained data is shown in Fig. 3(a). The time axis corresponds to the z-axis of the object and thus represents the depth information of the THz pulse. In Fig. 3(b) an exemplary THz pulse is shown at a fixed position of the line scan, which is indicated by the blue dotted line in Fig. 3(a). The line scan consists of many of these THz traces along the y-axis. When the THz pulse hits the surface of the sample a part of the THz pulse is reflected at the surface of the sample. Another part transmits through the sample until another interface occurs. Here, again a part of the radiation is reflected and transmitted, respectively. This process takes place until the entire radiation is absorbed or reflected back to the detector. A part of the radiation is also lost due to scattering in directions other than that of the detector. Depending on the structure of the surface and the layers beneath an amount between 7 and 40% of the incoming radiation is reflected back to the detector. THz pulses, which arise due to internal reflections within the sample are highlighted in red in Fig. 3(b). By plotting the time axis against a measurement line, the sub-surface structure of the sample can be represented. As shown in Fig. 3(a), at least two further levels of reflection below the skin surface of the mummy hand can be distinguished. As can be seen in the figure the gap between the layers increases in the region between 0 and 20 mm. During the aging process of the mummies the tissue dries out and simultaneously, air inclusions arise. The size of these air inclusions varies, which can be seen in the line scan. Additionally, a phase jump of the reflected THz radiation should occur in case of the reflection at an optically thicker medium. These phase jumps cannot be seen in Fig. 3(a). We attribute this behavior to some additional layers in between. These layers are so thin that the different THz pulses merge and cannot be readily distinguished anymore.

If the different layers, at which the THz pulse is reflected are so thin that the time for traveling through the layers is shorter than the THz pulse width, an overlap between the reflected THz pulses from different layers will occur. In this case it is not possible to detect all existing layers just by extracting the time position of the THz pulses directly. Further data analysis is required to extract the whole information about the layer structure of the sample. Therefore we used the sparse deconvolution algorithm described by Citrin and coworkers very recently^16^. Assuming that the measured signal is a convolution of the incoming THz pulse and the impulse response function of the sample, the algorithm aims to reconstruct the impulse response function of the sample using the assumption, that the impulse response function is approximately sparse. A more detailed description of the algorithm can be found in the methods section. With the algorithm it is possible to extract layer information out of very noisy measurement signals. This is important since the reflectivity of the mummies is quite low due their rough surface. Figure 3(c) shows the impulse response function of an exemplary deconvolved THz pulse, which is used for the tomographic reconstruction.

**Figure 4:**
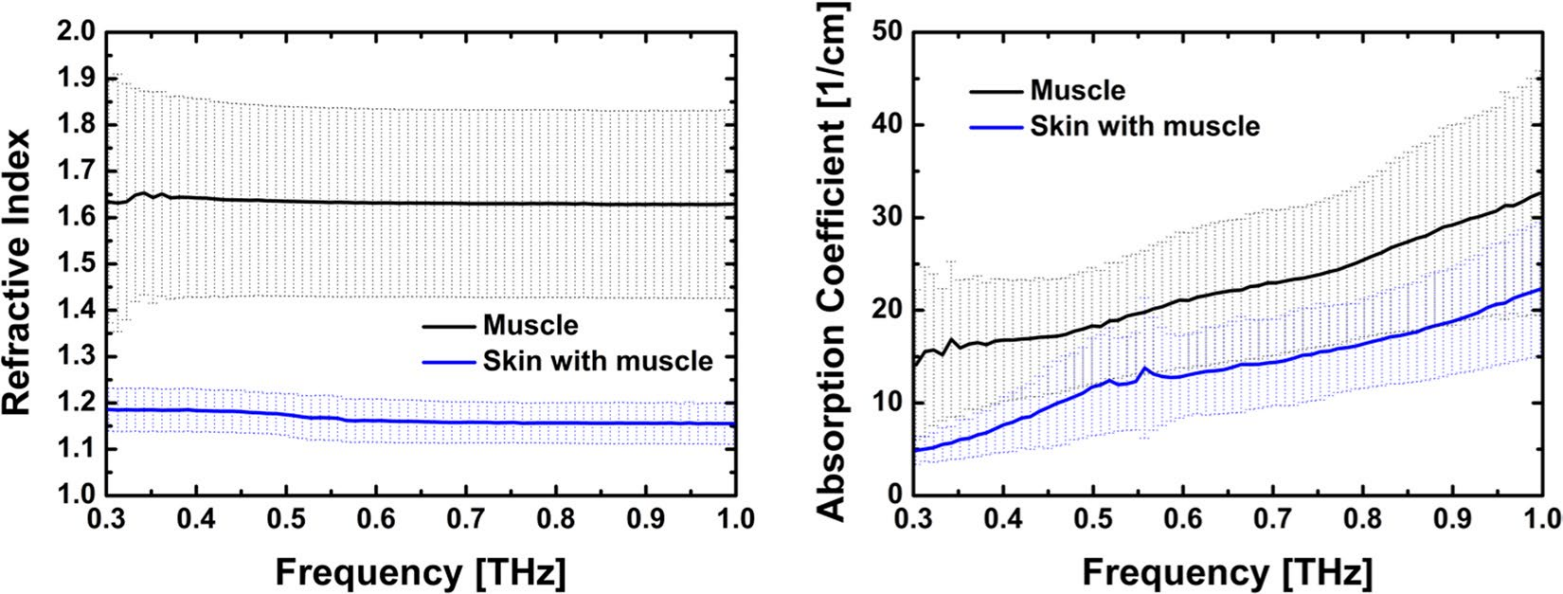
Dielectric properties of typical tissue structures in the investigated artificially mummified ancient tissue as well as in tissue from a modern mummification experiment. We have measured 5 (skin with muscle) respectively 10 (muscle) different positions on each sample and averaged them. The error arising in the spectroscopic measurements is mainly due to inhomogeneities in the samples.

To reconstruct the different layers with their real dimensions it is also necessary to know the refractive indices of the layer materials. Therefore, we have measured various samples of ancient mummified human tissue as well as from a modern human mummification experiment using a THz time-domain spectrometer in a transmission configuration. All samples were acquired and measured with institutional review board approval (BASEC N° 2017-00260) and belong to the collection of the Institute of Evolutionary Medicine, University of Zurich, Switzerland. The samples with the name ‘muscle’ originate from a modern mummification project (human lower leg)^36^. The two samples of this type had a size of 20 by 7 mm. They were measured at 5 different positions on each sample. The results are averaged after extracting the refractive index and absorption coefficient of each measurement with the commercially available Teralyzer software. In order to extract these parameters the thickness was measured at each position using a caliper. Furthermore, the Teralyzer software offers the possibility to find the correct thickness considering the Fabry-Perot-pulses in the measured transmission signal. The sample “skin with muscle” has a size of 15 by 15 mm and was taken from an ancient human mummified head of unknown origin. Again, 5 different points were measured and averaged. The estimated refractive indices and the absorption coefficients of all these samples are shown in Fig. 4.

### Tomographic reconstruction

**Figure 5:**
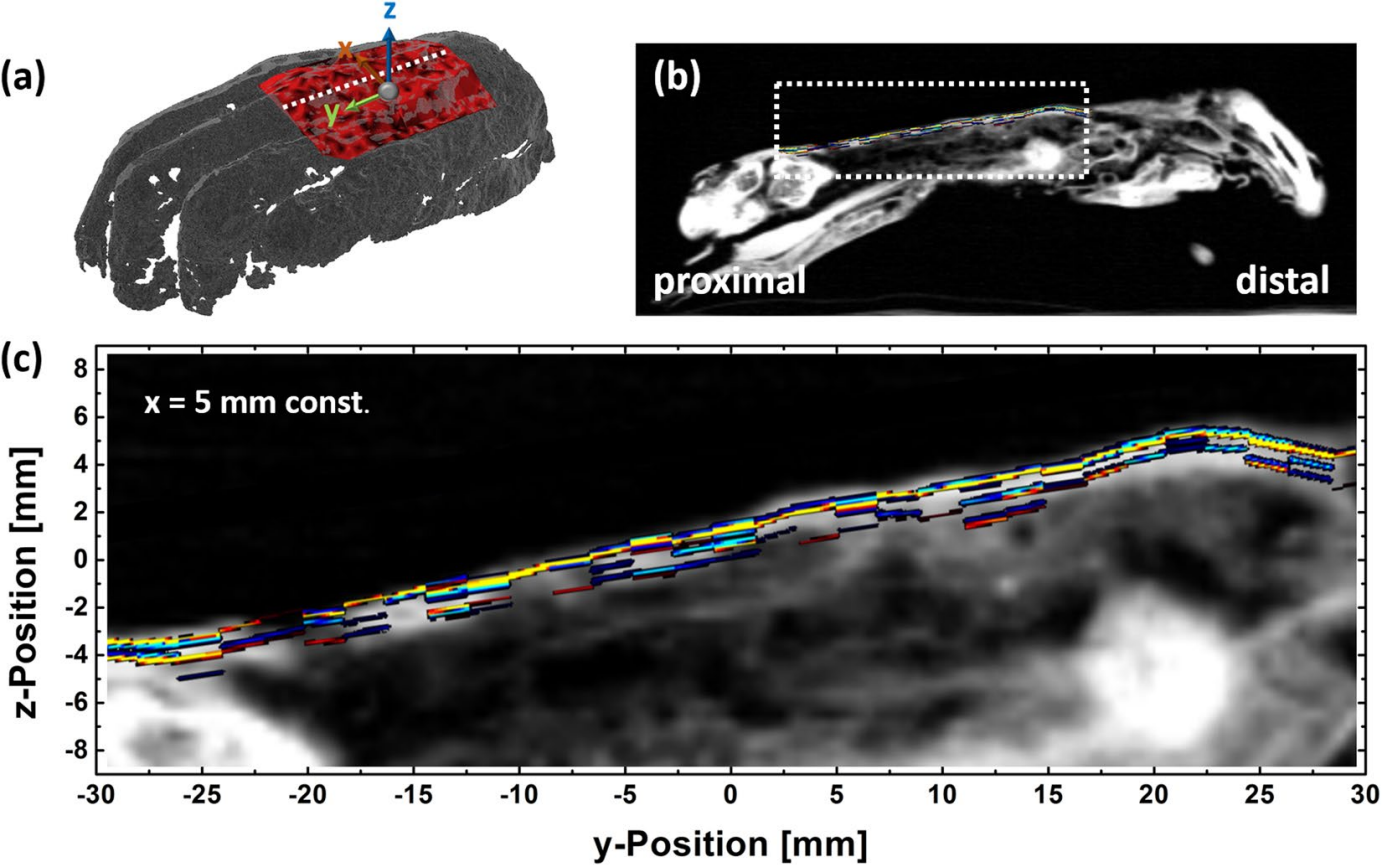
Comparison between reconstructed layers based on the THz measurement and a conventional CT scan. (**a**) The white dotted line in the 3D scan indicates the cross section at x = 5 mm, (**b**) THz measurement area in the CT scan. (**c**) Enlargement of the THz measurement area indicated by the white dotted rectangle in (**b**).

Figure 5 shows the results of a tomographic reconstruction of the THz time traces achieved with the robotic-based THz raster scan. With the sparse deconvolution algorithm it was possible to extract at least two layers beneath the skin. Each pixel represents the interface of two layers and has a lateral size of 2 mm. The color indicates the sign of the corresponding peak in the impulse response function. The intensity illustrates the magnitude. Thus, a change between red and blue colors indicates a phase jump of the THz wave at the interface between two layers. The amount of measurement points which contribute to each layer decreases with increasing depth.

To investigate and interpret the layer structure in more detail a cross section was reconstructed at a constant lateral position of x = 5 mm and a comparison with a conventional CT scan of the mummified hand is drawn parallel to the metacarpal bone (c.f. Fig. 5(b,c)). One can observe that the surface profile of the THz measurement is in a good agreement to the one of the CT scan. However, for internally layers it is hard to find coincidences since the resolution of the conventional CT scan is not high enough for a comparison. The THz data reveal more layers in the outer region of the hand than can be identified by the CT scan.

**Figure 6:**
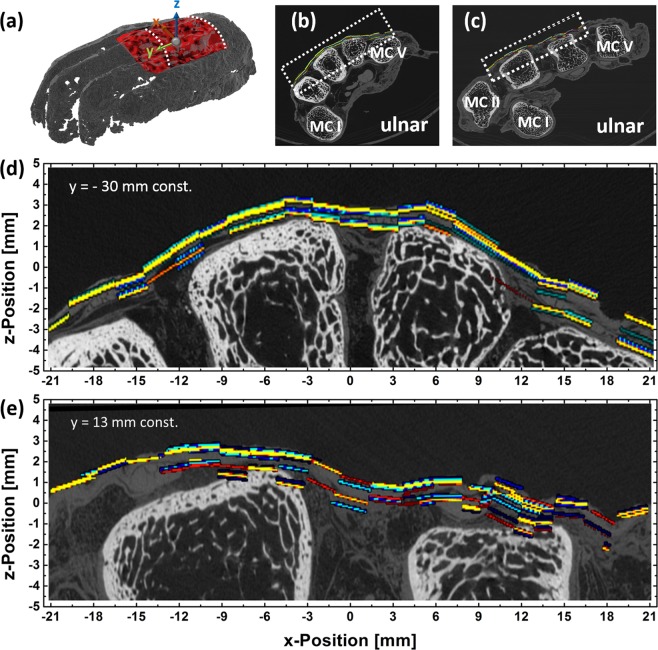
Comparison between reconstructed layers based on the THz measurement and a Micro- CT scan. (**a**) The white dotted lines in the 3D scan indicates the cross sections at y = 13 mm and y = −30 mm, (**b**) and (**c**) THz measurement region indicated in the CT scan. (**d**) Enlargement of the cross section at y = −30 mm indicated by the white dotted rectangle in (**b**), (**e**) Enlargement of the cross section at y = −13 mm indicated by the white dotted rectangle in (**c**).

For a more detailed comparison a micro-CT scan was performed. The results are summarized in Fig. 6. Two cross sections at constant y-positions are compared to the micro-CT scan. The first cross section at y = −30 mm, displayed in Fig. 6(d), shows a good agreement between the THz and the micro-CT data. The THz radiation penetrates the mummified hand until it hits the bone tissue of the hand. At several measurement points it is possible to identify different tissue layers above the bone corresponding to a different contrast in the micro-CT scan. Another cross section at y = 13 mm also exhibits multi-layer regions in the THz data which are again confirmed by the micro-CT scan.

## Discussion

In conclusion we have investigated a mummified hand with a robotic-based THz system. It is possible to extract information about the different skin layers up to a depth of 2 mm. In comparison to a conventional CT scan the depth resolution of the THz scan exceeds the resolution of the CT scan. To verify the results of the THz measurements an additional micro-CT scan was performed. It is in good agreement with the tomographic reconstruction of the THz data. The advantage of THz measurements over micro-CT scans is the fact that even comparatively large samples, like complete bodies, can be scanned. These would not fit into a conventional micro-CT scanner. Furthermore, THz measurements have the potential to identify embalming materials. This is made possible because the spectroscopic information is obtained simultaneously with the spatial information.

We have shown that THz measurements provide further information for pathological examinations in comparison to conventional CT scans. Thus, they are quite suitable as an additional tool to well established investigation techniques.

## Methods

### Robotic THz system

**Figure 7:**
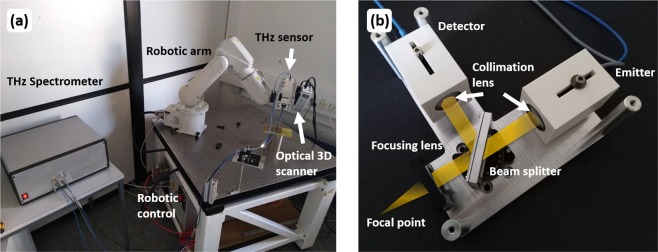
(**a**) Overview of the robotic based THz system, (**b**) Photo of the THz sensor head including a sketch of the THz beam path.

The robotic-based THz system, used for the measurements and shown in Fig. 7(a), consists of three different components. Detailed information can be found in^30^. The core of the system is the robotic arm ABB IRB 120 with six axes, a working range of 580 mm and a payload of 3 kg. Attached to this are a 3D scanner and a THz sensor head in reflection configuration. The 3D scanner is a commercially available fringe projection system (David SLS-2), which projects a varying stripe pattern onto the surface of the sample and detects the pattern with a camera in a defined angle. The 3D scanner system is calibrated by scanning a set of markers which is known to the system. This scheme is well established to reconstruct the surface profile of samples with curved surfaces^37^. Based on the data obtained with the 3D Scanner the measurement path for the THz sensor will be calculated. In order to do this it is necessary to map the 3D scanner’s coordinate system to that of the robotic arm. Therefore the position of a specially designed reference pattern in the robotic coordinate system is measured exactly with a tool tip mounted centrally on the robotic arm. Then the reference pattern is scanned with the 3D Scanner and a transfer matrix between the two different coordinate systems can be calculated. This process has to be done just once. The transfer matrix obtained in this way can be used for all further measurements.

In a next step the obtained surface profile of the sample is converted into the robotic coordinate system to calculate the measurement path of the THz sensor head. As a data format we choose a stl-file for the surface profile. This format describes the profile by dividing it into unstructured triangles with appropriate normal vectors. To calculate the position of the measurement points a two-dimensional user defined mesh is projected onto the surface. The z-coordinate for each point is obtained by tracing it in negative z-direction until an intersection with the surface occurs. The corresponding normal vector is calculated in an iterative process of averaging the normal vector of all triangles which are connected to the measurement point. After the x-, y- and z-coordinate and the normal vector is defined for each point this dataset is passed on to the simulation software of the robotic arm (ABB RobotStudio). This software determines the corresponding configuration for the six robotic axes to reach the measurement point. Simultaneously an automatic collision control is performed. The collision control checks if collisions between the robotic arm or the sensor head and the sample or the optical table would occur. If this is the case a simulation routine tries to find an alternative way for positioning the robotic arm. If no valid configuration can be found, the measurement point is deleted automatically. In this way it is possible to preserve the precious cultural assets from harm.

Finally the robotic-based THz measurement of the sample can start following the previously calculated measurement path. For the measurement of the mummified hand we used a THz sensor head with a silicon beam splitter to realize the reflection configuration. The THz beam path within this sensor head is shown in Fig. 7(b). The THz radiation emitted by the photoconductive antenna is first collimated by a high-density polyethylene (HDPE) lens. Afterwards the radiation passes through the silicon beam splitter positioned under an angle of 45 degree and is focused by a second HDPE lens onto the sample. The focal length of the lens is 50 mm. The reflected THz radiation passes this lens again and is reflected at the silicon beam splitter towards the THz receiver antenna. In front of the antenna a third HDPE lens focuses the THz radiation onto the antenna. The lateral scanning resolution of this sensor head, given by the diameter of the focal point, is 1.6 mm. The bandwidth for a reflection at a metal reference is 2 THz.

The THz sensor head was driven by the TeraWave system developed at the Heinrich-Hertz-Institute in Berlin^25^. The entire system and the data acquisition are operated by a Python-based software. For the measurement 100 waveforms were averaged per measurement point resulting in a measurement time of about 20 s per point including the time for the movement between two points.

### Algorithm for the tomographic reconstruction

To convert the time axis of the THz pulses into a spatial information it is necessary to extract the single THz pulses reflected at each layer. Since the measured signal is a convolution of the incoming THz pulse and the impulse response function (IRF) of the sample, a sparse deconvolution algorithm is used which is described in detail in 16. It assumes that the impulse response function of the sample’s reflection is approximately sparse, i.e. that it is non-zero only at very few time positions. These positions should correspond to the interfaces of the various layers that the THz pulse is reflected from. Based on this approximation, an optimization algorithm tries to find a sparse solution to the IRF. In simplified terms, the algorithm tries to minimize the l0 norm of the difference between measured time-domain trace and calculated time-domain trace based on the sparse transfer function and a reference trace. To account for noise, a user-defined regularization parameter is added. Since minimizing the l0 norm is computationally very hard, the algorithm approximates it with the l1 norm which is much easier to compute. The algorithm is of the iterative shrinking type, developed in^38^. As described in^16^ the deconvolution algorithm can be extended to take frequency-dependent absorption and pulse spreading into account.

The temporal positions of the non-zero values in the resulting IRF are then assigned to the individual layers according to their temporal order. To obtain the actual 3D-coordinate of such a point of a layer, the spatial measurement coordinate is translated along its normal vector by the distance corresponding to its IRF peak position. The distance calculation takes into account the different velocities of light in the individual layer materials. Hence, the algorithm relies on prior knowledge of the refractive indices of the materials involved.

Applying this algorithm to all data points transforms the temporal into spatial information and thus results in a tomographic reconstruction of the sample based on the THz time-domain data.

### CT scans

CT scans were performed at the Balgrist University Hospital, Zurich, Switzerland, using a multislice CT unit (Brilliance 40, Philips Medical Systems, Amsterdam, The Netherlands). The specimen was placed in the scanner in axial orientation with the palmar side facing down. Acquisition parameters included tube voltage of 120 kVp, fixed exposure setting of 120 mAs. Axial, coronary and sagittal reconstructions were performed using the CT unit’s workstation with convolution kernel D for filtered back projection. Parameters included slice thickness of 2 mm, a 512 × 512 matrix and a data collection diameter of 149 mm.

### Micro-CT scans

Micro-CT scans were performed at Scanco medical AG, Brüttisellen, Switzerland, a manufacturer of micro-CT scanners, using an XtremeCT II unit. The specimen was again placed in the scanner in axial orientation with the palmar side facing down. Acquisition parameters included tube voltage of 68 kVp, and exposure of 132 mAs. Axial reconstructions were performed using the manufacturer’s reconstruction software (version V6.2-4) with an isotropic voxel size of 20 μm.

## Data Availability

The data and algorithm support the findings of this study are available from the corresponding author upon reasonable request.

## References

[1] Aufderheide AC (2000). Progress in soft tissue paleopathology. JAMA.

[2] Ikram, S. & Dodson, A. *Mummy in Ancient Egypt: Equipping the Dead for Eternity*. (Thames and Hudson, 1998).

[3] Cockburn, T. A., Cockburn, E. & Reyman, T. A. *Mummies, Disease and Ancient Cultures* (Cambridge University Press, 1998).

[4] Lynnerup N (2007). Mummies. Yearb. Phys. Anthr..

[5] Thompson RC (2013). Atherosclerosis across 4000 years of human history: The Horus study of four ancient populations. Lancet.

[6] Beckett RG (2014). Paleoimaging: A review of applications and challenges. Forensic Sci. Med. Pathol..

[7] Lynnerup N (2010). Medical imaging of mummies and bog bodies - A mini-review. Gerontology.

[8] Rühli FJ, Chhem RK, Böni T (2004). Diagnostic paleoradiology of mummified tissue: interpretation and pitfalls. Can. Assoc. Radiol. J..

[9] Lynnerup N (2009). Methods in mummy research. Anthropol. Anzeiger.

[10] Lynnerup N, Rühli F (2015). Short Review: The Use of Conventional X-rays in Mummy Studies. Anat. Rec..

[11] Hu BB, Nuss MC (1995). Imaging with terahertz waves. Opt. Lett..

[12] Mittleman DM (1999). Recent advances in terahertz imaging. Appl. Phys. B Lasers Opt..

[13] Jepsen PU, Cooke DG, Koch M (2011). Terahertz spectroscopy and imaging - Modern techniques and applications. Laser Photonics Rev..

[14] Peiponen, K. E., Zeitler, J. A. & Kuwata-Gonokami, M. *Terahertz Spectroscopy and Imaging*. 171, (Springer Berlin Heidelberg, 2013).

[15] Mittleman DM, Hunsche S, Boivin L, Nuss MC (1997). T-ray tomography. Opt. Lett..

[16] Dong J, Wu X, Locquet A, Citrin DS (2017). Terahertz Superresolution Stratigraphic Characterization of Multilayered Structures Using Sparse Deconvolution. IEEE Trans. Terahertz. Sci. Technol..

[17] Redo-Sanchez A (2016). Terahertz time-gated spectral imaging for content extraction through layered structures. Nat. Commun..

[18] Scheller M, Jansen C, Koch M (2009). Analyzing sub-100-μm samples with transmission terahertz time domain spectroscopy. Opt. Commun..

[19] Krügener K (2015). terahertz meets sculptural and architectural art: evaluation and conservation of stone objects with t-ray technology. Sci. Rep..

[20] Hintzsche H (2013). Terahertz Radiation at 0.380 THz and 2.520 THz Does Not Lead to DNA Damage in Skin Cells *In Vitro*. Radiat. Res..

[21] Fischer BM, Walther M, Jepsen PU (2002). Far-infrared vibrational modes of DNA components studied by terahertz time-domain spectroscopy. Phys. Med. Biol..

[22] Fukunaga, K. *THz Technology Applied to Cultural Heritage in Practice*, 10.1007/978-4-431-55885-9 (Springer Japan, 2016).

[23] Cosentino A (2016). Terahertz and Cultural Heritage Science: Examination of Art and Archaeology. Technologies.

[24] Inuzuka M, Kouzuma Y, Sugioka N (2017). Investigation of Layer Structure of the Takamatsuzuka Mural Paintings by Terahertz Imaging Technique. J. Infrared, Millimeter, Terahertz Waves.

[25] Vieweg N (2014). Terahertz-time domain spectrometer with 90 dB peak dynamic range. J. Infrared, Millimeter, Terahertz Waves.

[26] Nüßler, D., Heinen, S., Sprenger, T., Hübsch, D. & Würschmidt, T. T-SENSE a millimeter wave scanner for letters. In *Proc. SPIE 8900, Millimetre Wave and Terahertz Sensors and Technology VI* 89000M, 10.1117/12.2029172 (2013).

[27] Mittleman DM, Hunsche S, Boivin L, Nuss MC (1997). T-ray tomography. Opt. Lett..

[28] Hunsche S, Mittleman DM, Koch M (1998). M. C. N. New Dimensions in T-Ray Imaging. IEICE Trans. Electron..

[29] Brucherseifer M, Haring Bolivar P, Klingenberg H, Kurz H (2001). Angle-dependent THz tomography – characterization of thin ceramic oxide films for fuel cell applications. Appl. Phys. B.

[30] Stübling E (2017). A THz Tomography System for Arbitrarily Shaped Samples. Journal of Infrared, Millimeter, and Terahertz Waves.

[31] Öhrström L (2015). Terahertz Imaging Modalities of Ancient Egyptian Mummified Objects and of a Naturally Mummified Rat. Anat. Rec..

[32] Öhrström L, Bitzer A, Walther M, Rühli FJ (2010). Technical note: Terahertz imaging of ancient mummies and bone. Am. J. Phys. Anthropol..

[33] Cosentino A, Leona M, Mininberg DT (2011). Investigating the use of terahertz pulsed time domain reflection imaging for the study of fabric layers of an Egyptian mummy. J. Eur. Opt. Soc. - Rapid Publ..

[34] Jackson JB (2014). Terahertz pulse imaging in archaeology. Front. Optoelectron..

[35] Kreissl Lonfat, B. M., Kaufmann, I., Bouwman, A., May, H. & Frater, N. Code of Ethics. 1–10 (2014). Available at: http://www.iem.uzh.ch/institute/iemcodeofethics/Code_of_Ethics_IEM_2014.pdf. (Accessed: 17th December 2018).

[36] Papageorgopoulou C, Shved N, Wanek J, Rühli FJ (2015). Modeling Ancient Egyptian Mummification on Fresh Human Tissue: Macroscopic and Histological Aspects. Anat. Rec..

[37] Rocchini C, Cignoni P, Montani C, Pingi P, Scopigno R (2001). A low cost 3D scanner based on structured light. Comput. Graph. Forum.

[38] Elad M, Matalon B, Shtok J, Zibulevsky M (2007). A wide-angle view at iterated shrinkage algorithms. Proc. SPIE.

